# Optimizing blood–brain barrier permeation through deep reinforcement learning for *de novo* drug design

**DOI:** 10.1093/bioinformatics/btab301

**Published:** 2021-07-12

**Authors:** Tiago Pereira, Maryam Abbasi, José Luis Oliveira, Bernardete Ribeiro, Joel Arrais

**Affiliations:** CSUC/DEI, University of Coimbra, Coimbra 3030-290, Portugal; IEETA/DETI, University of Aveiro, Aveiro 3810-193, Portugal; CSUC/DEI, University of Coimbra, Coimbra 3030-290, Portugal; IEETA/DETI, University of Aveiro, Aveiro 3810-193, Portugal; CSUC/DEI, University of Coimbra, Coimbra 3030-290, Portugal; CSUC/DEI, University of Coimbra, Coimbra 3030-290, Portugal

## Abstract

**Motivation:**

The process of placing new drugs into the market is time-consuming, expensive and complex. The application of computational methods for designing molecules with bespoke properties can contribute to saving resources throughout this process. However, the fundamental properties to be optimized are often not considered or conflicting with each other. In this work, we propose a novel approach to consider both the biological property and the bioavailability of compounds through a deep reinforcement learning framework for the targeted generation of compounds. We aim to obtain a promising set of selective compounds for the adenosine $A_{2A}$ receptor and, simultaneously, that have the necessary properties in terms of solubility and permeability across the blood–brain barrier to reach the site of action. The cornerstone of the framework is based on a recurrent neural network architecture, the Generator. It seeks to learn the building rules of valid molecules to sample new compounds further. Also, two Predictors are trained to estimate the properties of interest of the new molecules. Finally, the fine-tuning of the Generator was performed with reinforcement learning, integrated with multi-objective optimization and exploratory techniques to ensure that the Generator is adequately biased.

**Results:**

The biased Generator can generate an interesting set of molecules, with approximately 85% having the two fundamental properties biased as desired. Thus, this approach has transformed a general molecule generator into a model focused on optimizing specific objectives. Furthermore, the molecules’ synthesizability and drug-likeness demonstrate the potential applicability of the *de novo* drug design in medicinal chemistry.

**Availability and implementation:**

All code is publicly available in the https://github.com/larngroup/De-Novo-Drug-Design.

**Supplementary information:**

Supplementary data are available at *Bioinformatics* online.

## 1 Introduction

Drug development, the process of placing new drugs into the market, is a lengthy and costly task that, in practice, can take around ten years and cost 2.6 million USD (Chan *et al.*, 2019). The generation of an initial set of promising molecules (lead compounds) can speed up this complex process. These lead compounds are usually discovered using iterative organic synthesis and screening assays with a high failure rate (Hughes *et al.*, 2011). In recent years, computational methods have contributed significantly to development in all areas, and drug discovery has not been an exception. Remarkably, given the large amounts of available data, methods based on machine learning (ML) and deep learning (DL) have been integrated into this field to generate and optimize molecules. The goal is to increase effectiveness and reduce the resources spent in this process (Chen et al., 2018).

In the generation of potential drug compounds, one must consider the molecular characteristics such as binding affinity towards specific targets as well as the properties associated with its bioavailability. One crucial parameter is the optimization of drugs’ permeability through the Blood–Brain Barrier (BBB). Even in protocols that generate molecules designed to act on the brain, it is often ignored or treated lightly due to biological membrane’s restrictiveness. Consequently, identifying properties in the compounds associated with their BBB permeability is a challenge that has not been tackled as desired in the drug discovery field.

There are several approaches in the literature for the generation and optimization of key molecular properties. Targeted generation requires an evaluator of the newly generated compounds to conduct the training process towards the most promising solutions. Therefore, these works typically integrate a quantitative structure-activity relationship (QSAR) model into their frameworks. QSAR modelling allows predicting the characteristics of interest on the molecular structure of the compounds. The most common descriptors for this model are the extended connectivity fingerprint (ECFP) (Liu et al., 2019). However, QSARs can be built using neural networks to extract the descriptors directly from the simplified molecular-input line-entry system (SMILES) or graphs of the compounds (Duvenaud et al., 2015; Popova et al., 2018). Then, it’s possible to use the QSAR model’s outcome to guide the process of generation and optimization of molecules. evolutionary algorithms (EAs) (Devi *et al.*, 2015) and reinforcement learning (RL), have been applied to conduct in this task (Sanchez-Lengeling and Aspuru-Guzik, 2018; Ståhl et al., 2019).

Nicolaou et al. (2009) employed evolutionary techniques to reconstruct atoms and bonds in molecules described through graphs. This manipulation of the original molecules aimed to produce molecules optimized in terms of one or more molecular objectives of interest. DL models, together with RL, are the alternative approach to produce and optimize compounds properties. Sanchez-Lengeling et al. (2017) have explored the integration of a generative adversarial network (GAN) with RL to perform a biased molecular generation. An alternative was implemented by You et al. (2018) applying a graph-based convolutional network (GCPN) with RL for targeted generation of molecules. Moreover, Popova *et al.* (2018), Olivecrona et al. (2017) and Liu *et al.* (2019) have adopted the policy-based RL method named REINFORCE with SMILES notation and recurrent architectures for the targeted generation of molecules. Zhou et al. (2019) have implemented a value-based Reinforcement Learning approach to design molecules with specific properties, formalizing the problem through a Markov decision process (MDP). Also, several types of policy-based RL setups have been implemented, namely, proximal policy optimization where the molecule is generated with the addition of a new bond in each step in order to optimize the affinity-related properties for specific targets (Khemchandani et al., 2020). Ståhl *et al.* (2019) implemented an RL approach based on an actor-critic model for the generation of novel molecules. They optimized properties such as the partition coefficient (logP), polar surface area (PSA) and molecular weight (MW) starting from a set of lead molecules. Recently, Deng *et al.* (2020) implemented a solution with a multi-objective reward function, designed to bias the generation towards molecules with both sufficient opioid antagonistic effect and ability to cross BBB and stay in the brain. However, one of the main problems with this approach is that BBB permeation is not being considered directly but instead by basic properties that cannot describe all the complexity of the mechanisms of drugs passing through BBB. There are several mechanisms, either passive or active and non-obvious interactions responsible for controlling molecules’ permeation across the BBB. Hence, the inability of computational generative methods to combine the optimization of biological and psycho-chemical properties with BBB permeation is one major critique in this work.

To maximize the chance of finding interesting hits for a given target, generated drug candidates must possess biological affinity as well as absorption capacity and permeability across the body membranes. Although most current computational solutions concentrate on one aspect or the other, drug design protocols should provide the same importance to the drug–target interaction factor and the drug’s potential to reach the site of action.

This article proposes a framework to automatically generate new molecules from scratch. Specifically, the aim is to perform a broader exploration of the chemical space, to identify molecules with biological affinity for the desired target, bioavailability and novelty comparing with the existing solutions. This work’s particular interest is the optimization of two molecular properties simultaneously, one being a drug–target affinity and the other the bioavailability at the central nervous system.

The proposed framework is a deep RL model that generates molecules with high affinity to inhibit adenosine A2A receptor ($A_{A2A}R$) (Chen *et al.*, 2013), and that can also pass through the blood–brain barrier. This target is a type of G Protein-Coupled Receptor (GPCR) that is involved in the treatment of conditions such as insomnia, pain, depression, Parkinson’s disease, cardiovascular diseases and inflammatory disorders (Chen *et al.*, 2013). Therefore, identifying effective $A_{A2A}R$ antagonists is a task of utmost importance in computational drug discovery. Moreover, it introduces specific procedures to enhance diversity and novelty in the newly generated compounds. To prevent the repetitive generation of molecules, it creates a dynamic memory cell to penalize the reward whenever the set of molecules’ diversity decreased during exploring chemical space. Finally, it introduces the multi-objective optimization mechanism joined with RL to optimize the compounds’ properties simultaneously.

## 2 Materials and methods

**Fig. 1.**
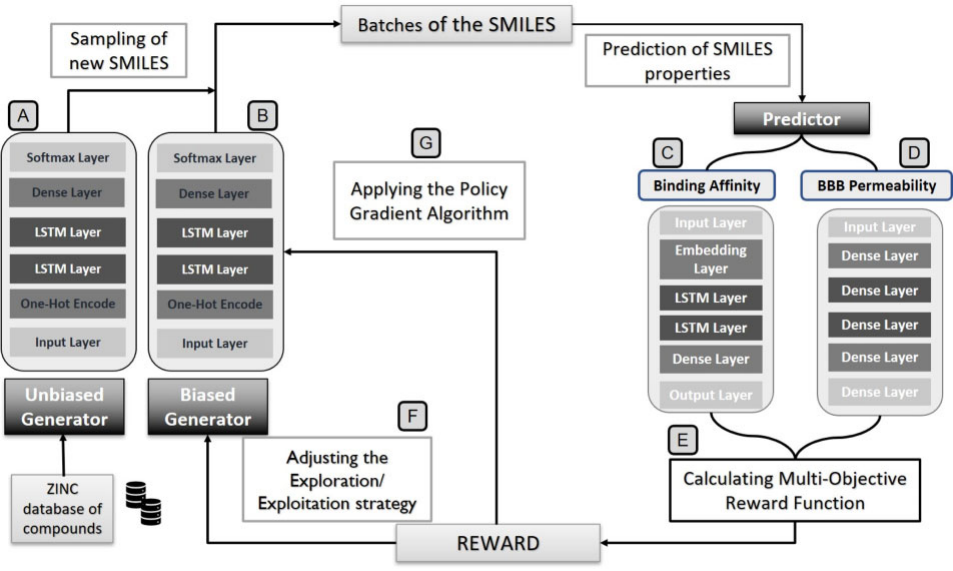
The general framework contains 4 DL modules: an unbiased Generator (**A**), a biased Generator (**B**) which sharing the same architecture and two QSAR models for predicting the binding affinity (**C**) and BBB permeation (**D**). The DL modules are interconnected by a policy-based Reinforcement Learning approach (**G**) applied with a particular exploration/exploitation strategy (**F**) based on a multiobjective reward function (**E**)

The general overview of the proposed framework has been shown in Figure 1. The framework contains a Generator based on recurrent neural networks (RNNs) and two Predictors for the two considered molecular properties. The applied RL methodology is based on a policy-gradient algorithm. For the RL application, we create a copy of the initially trained Generator. One of the models is kept uninvolved, the unbiased Generator (Fig. 1A), and the other is updated throughout the RL process, the biased Generator (Fig. 1B). Thus, we integrate the two generators interchangeably sharing the same architecture but with different internal parameters: the biased Generator is involved with exploration. The unbiased Generator is focused on exploitation. The two predictors conduct the updates in the biased Generator weights considering the maximization of the measure drug–target binding affinity, and the BBB permeability.

The implementation of this framework divides into two phases. In the first phase, Supervised Learning (SL) is employed to train the unbiased Generator and both Predictors. The binding affinity Predictor for $A_{A2A}R$ (Fig. 1C) is built with SMILES as input data. The BBB permeability Predictor (Fig. 1D) is implemented using the Extended Connectivity Fingerprint (ECFP) (Rogers and Hahn, 2010). In the second step, RL is applied to retrain the biased Generator. The Predictors worked as guides of this training process since the combination of the rewards from each drive the Generator to explore new chemical spaces. The maximization of the combined rewards would lead to the optimization of the desired molecular properties. Multi-objective optimization is integrated through the construction of the reward function that considers the two competing objectives fairly. Furthermore, throughout the RL training process, the exploration/exploitation dilemma has been considered since the compounds’ diversity, and validity is vital.

### 2.1 Generator

The Generator learns the basic rules for building molecules through SMILES notation. The model architecture (described in Fig. 1A and B) consists of an input layer converted to one-hot encoded vectors, followed by two LSTM layers with 256 units, and a densely connected layer with 43 units before the output layer with Softmax activation. In this regard, it is possible to associate each token with the respective probability of being selected. Between each LSTM layer, a dropout regularization is applied to minimize the learning inter-dependency and maximize the model’s generalization ability. Therefore, at each step, the prediction of the next token is made, taking into account the molecule hitherto built. The Teacher Forcing methodology is implemented throughout this process. Thus, at each step, the set of correct tokens of the molecule is given as input instead of the model’s previous predictions to accelerate convergence (Gupta et al., 2018). The loss function is the negative log-likelihood ratio between the correct and competing tokens, as depicted in Equation 1. As a consequence, the probability of choosing the correct token is maximized at each step.

(1) $$J(\theta )=-\frac{1}{T}\sum_{t=1}^{T}\left[y_{t}\;\text{log}\;{\hat{y}}_{t}+(1-y_{t})\;\text{log}(1-{\hat{y}}_{t})\right].$$

Data is divided into batches of 16 SMILES, the model is trained during 25 iterations, and the optimizer applied to update the weights is Adam with a learning rate of 0.001. Moreover, the gradient module is limited to 3 through gradient clipping to guarantee the value’s stability during training (Pascanu et al., 2013). After the training step, the Generator samples new molecules by predicting its structure token-by-token. Therefore, the next atom or bond integrating the molecule heavily depends on the structure synthesized up to that point. Finally, molecules are syntactically validated by the RDKit molecule sanitizer (Landrum, 2019).

### 2.2 Predictor

Predictor models are a fundamental part of this framework as they perform the molecule’s evaluation and, consequently, the process of exploring chemical space that is conducted by the assigned rewards. The fundamental idea is to implement two QSAR models: one estimate biological affinity for $A_{A2A}R$ and the other predict BBB permeability for molecules. The methodology applied for each QSAR implementation depends on the constraints imposed by the available data. The first Predictor is implemented as a descriptor-free QSAR model since it was possible to find datasets with a reasonable number of compounds to predict the molecules’ biological affinity. The proposed QSAR model, depicted in Figure 1C, employs only SMILES strings as the molecular descriptor to train the neural network based on recurrent architecture. SMILES are encoded and then given as input to the architecture formed by an embedding layer (where each token is converted into a vector of 128 elements), two GRU layers (128 units) and a densely connected layer (128 units activated by ReLU) that connects to the output layer (one unit with linear activation). Using the SMILES notation makes it possible to keep all information about the molecules’ structure and chirality inside a string. This descriptor implies low computational cost to obtain and not excessive overhead for the training process of the QSAR. In addition, SMILES is a sequential representation of molecules, and it is very well suited to GRU/LSTM cells. Therefore, it is used as input data with varying lengths. More importantly, the RNN architecture can capture the most prominent parts of the compounds to build a reliable QSAR (Chakravarti and Alla, 2019).

For the BBB QSAR, depicted in Figure 1D, we followed a different approach due to the constraints associated with predicting the ability of molecules to cross the membrane. Firstly, the main obstacle is related to the dataset: the scarcity of examples and class imbalance. In DL, it is essential to support the implementation of models in balanced datasets and with enough examples to avoid overfitting. Specifically, the datasets used to predict BBB permeability are often formed with more examples of molecules of the permeable class (BBB+) than of the class that cannot cross the membrane (BBB-). For that reason, one of the most frequent undesirable outcomes of these models is the high false-positive rate (Wang et al., 2018). Secondly, another limitation for implementing these models is the high number of features needed to describe the process’s complexity (Almutairi *et al.*, 2016).

The proposed approach aims to find solutions for these drawbacks in a coordinated way. The goal is to implement a binary classification model by testing two methods: the first is based on RNNs and SMILES notation. The second is based on a deep fully connected neural network (DFCNN) employs ECFP as the only molecular descriptor. The scarcity of data is the most challenging problem to solve. Nonetheless, we gathered a collection of compounds from other works, and it was possible to achieve a dataset of 4534 molecules, which is an acceptable size for a DL model.

**Fig. 2.**
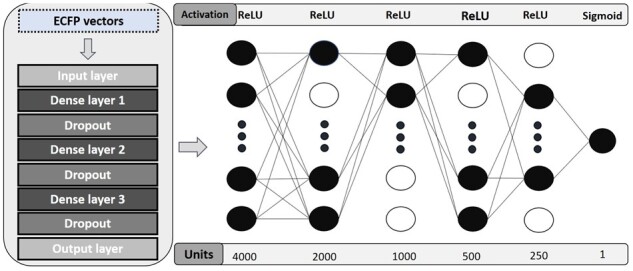
Predictor architecture for the BBB permeability: The ECFP vectors feed five successive dense layers activated by ReLU function to which drop-out is applied. A sigmoid function activates the output layer to guarantee outputs close to 0 (non-permeable) and 1 (permeable)

However, as explained, these datasets are unbalanced, and only 750 out of 4534 were of the BBB- class. The proposed solution is to apply oversampling techniques to balance the ratio between the two classes. Alsenan *et al.* (2020) have employed Synthetic Minority Oversampling Technique (SMOTE) as the re-sampling method to balance the class imbalance. This work will test the SMOTE and Adaptive Synthetic (ADASYN) sampling algorithms to balance classes. Each ECFP vector is a descriptor that incorporates molecules’ structural and functional characteristics in a bit string format and is commonly applied for tasks such as virtual screening, similarity searching and clustering. In the case of circular fingerprints such as ECFPs, which are obtained by implementing Morgan’s algorithm, molecular structures are represented employing circular atom neighbourhoods and, sequentially, assigning numbers to each atom of each molecular structures. For the implementation of the BBB QSAR, the vectors were created with 2048 elements and with radius 2, using the RDKit tool. Figure 2 shows the DFCNN contained five fully connected hidden layers (with 4000, 2000, 1000, 500 and 250 neurons, respectively) activated by Rectified Linear Unit (ReLU). The output layer had a sigmoid activation, and the binary cross-entropy was selected as the loss function since it is a classification problem with two classes.

For the Predictor implementations, the best hyper parameters are determined using a grid-search strategy. Moreover, the performance of both QSAR models is performed using five-fold cross-validation to split the data and prevent unwanted overfitting. The data is divided into 85% for training/validation and 15% for testing. Then, the training/validation data are divided into five folds to train an equal number of models and in each fold, SMILES is randomly divided into 85% of the training data 15% for validation. The robustness of models is evaluated with an external test set. Moreover, to avoid a too-tight adjustment of the models to the training data, the early stopping technique is applied. In this way, it is possible to arbitrate a large number of epochs that the training process will end when the performance of the model does not improve in the validation subset.

### 2.3 Reinforcement learning

The followed approach, represented in Figure 1G, is implemented with the REINFORCE algorithm (Williams, 1992), and the goal is for the Generator to learn the actions that must take at each state during the process of synthesizing new molecules to optimize the desired properties. This learning process is based on a Markov Decision Process. It’s necessary to have an agent, the surrounding environment with several possible states and a set of actions that the agent can select. The dynamics of this process imply that, at each step, the agent interacts with the environment by choosing one possible action. In the next step, a reward is attributed to the agent, taking into account the previous state’s action. A new state of the environment is presented to the agent to repeat the process. The policy is the tool that performs the mapping between the current state to the distribution of probabilities for choosing the next action. This process’s objective is approximating the policy that ensures the maximum possible reward (François-Lavet et al., 2018).

In general, this formulation can be represented using the Equation 2. On that account, lower rewards correspond to incorrect behaviour/policy, whereas higher rewards indicate that the behaviour/policy is evolving in the right direction.

(2) $$R_{t}=\sum_{k=0}^{T}\gamma^{k}r_{t+k+1}.$$

where *R_t_* is the return, *t* is the time step, *T* is the final time step and *γ* is a discount factor. It is a parameter that ranges from 0≤γ<1 and determines how much the future reward worths in the present (Sutton and Barto, 1998). The process of generating molecules based on SMILES notation can be adapted for an MDP. The Generator represents the agent, and its weights correspond to the policy. The choice of each token during the construction of SMILES can be seen as each action’s choice. Furthermore, the successive intermediate states of the molecules being generated correspond to the successive states of the environment presented to the agent and based on which the agent must decide what should be the next token/action.

The policy is the cornerstone of RL dynamics. The weights of the Generator will be updated based on the gradient of a scalar performance measurement (J(θ) in Equation 3) with respect to the policy parameters. The goal is to maximize this performance objective so that their updates approximate gradient ascent in *J*:

(3) $$\theta_{t+1}=\theta_{t}+\alpha \nabla J(\theta_{t}),$$

where *t* represents the time step, *θ* the policy parameters, *α* the learning rate and $\nabla J(\theta_{t})$ is the estimation, through its expectation, of the gradient of the performance measure with respect to *θ_t_* (Sutton and Barto, 1998). Equation 4 represents the REINFORCE update obtained by using this sample to instantiate our generic stochastic gradient ascent algorithm (Equation 3).

(4) $$\theta_{t+1}=\theta_{t}+\alpha \gamma^{t}R_{t}\nabla ln\pi (A_{t}|S_{t},\theta_{t}).$$

As it is explained in Section 2.5, it is through the manipulation of the reward function (*R_t_*) that we will take into account the two properties to be optimized.

### 2.4 Learning process

The process of fine-tuning the Generator is based on the repetitive sampling of new molecules. SMILES are generated in batches of 10 elements. Both Predictors assign a reward to the molecules that will be higher, as the better the molecular properties fit to what is desired. Afterwards, each molecule is ‘decomposed’ in the respective tokens that compose it to analyze the probabilities associated with the choice of each action. Based on the obtained reward, the loss function shows to the policy whether the chosen actions in those specific states of the molecule generation process are encouraged (the reward obtained was satisfactory) or are discouraged (the reward obtained was unsatisfactory) in future visits to the same state. After performing this process for all molecules present in the batch, we obtain a cumulative loss (Equation 5), with which the gradient descent method is applied.

(5) $$J(\theta_{t})=-\frac{1}{n}\sum_{i=1}^{\left|S\right|}\sum_{j=1}^{\text{length}(s_{i})}R_{i}\cdot \gamma^{i}\cdot \ln(p(s_{j}|s_{0}\ldots s_{j-1},\theta )).$$

The gradient of the loss function is calculated with regard to the policy, i.e. in relation to the weights of the Generator. Typically, the negative gradient of a function at a given point will be a vector tangential to the surface points in the direction where the function decreases most rapidly (Ruder, 2016). In this case, the direction in the parameter space (indicated by the gradient) that most minimizes the loss function is the direction corresponding to the choice of actions that provide the greatest reward during the molecule generation process. After several cycles of batches of molecules, the parameters of the Generator should be biased to choose the actions that maximize the reward and, consequently, the Generator should be biased towards molecules with high affinity for $A_{A2A}R$ and potential to cross the BBB.

### 2.5 Multi-objective reward function

The design of the reward function is crucial for molecular optimization, particularly when there are multiple objectives to be integrated. The aim is to ensure that the importance assigned to each objective is fairly distributed so that both the biological affinity for the receptor and the permeability across BBB are skewed in the desired direction in the newly generated molecules. Thus, we develop several possible solutions in order to build the approximated Pareto diagram. This diagram is widely used to evaluate solutions in multi-objective reinforcement learning problems, as it illustrates compromised solutions among the objectives (Nguyen, 2018). The objective is to approximate the true Pareto front since it contains the solutions that dominate all the others. A solution dominates another if it is superior on at least one objective, and at least, equal on all other objectives (Ehrgott, 2005). Therefore, considering that it is mathematically and computationally challenging to find the true Pareto front, we calculate the approximation set. In this work, we compute two approximate Pareto fronts by applying two scalarization techniques that transform the vector with two rewards into a single numeric.

The first applied method is linear scalarization, which combines the two objectives. A weight is assigned to each of the objectives. Then a weighted average is calculated, which enables to obtain a unique value that reflects the influence of the two objectives (Van Moffaert et al., 2013). This advantage of this method is straightforward to implement and the possibility of adopting a similar RL configuration compared to the single-objective optimization. Nonetheless, this scalarization technique has the limitation of finding only policies that rely on the convex zones of the Pareto optimal set (Ehrgott, 2005). Note that the weight assigned to each objective can vary between 0 and 1, and the only condition is that the sum of both objective values must be 1.

The second method is a non-linear scalarization technique, based on Chebyshev metric (Ehrgott, 2005). In this case, before starting the RL process, a utopian maximum of the reward is defined for each objective. Since both objectives are normalized between 0 and 1, the maximum possible is 1. Then, at each step, the distance between the obtained reward and the previously defined maximum is calculated (weighed by a factor between 0 and 1). The selected objective to be optimized is the one with the largest distance to the optimum point. This technique is non-linear because it uses only one reward at each step and allows solutions to be sought in all locations of the Pareto front (Brys *et al.*, 2013).

### 2.6 Exploration/exploitation dilemma

As represented in Figure 1F, it is essential to control the exploration/exploitation dilemma when employing RL to ensure that the chemical spaces of interest are discovered. For that reason, it is necessary to balance exploration (which seeks to acquire more information about the environment so that future actions can be more advantageous) with exploitation (which privileges the choice of actions based on current knowledge) (Sutton and Barto, 1998). This trade-off is controlled in three phases throughout this work.

First, a Softmax activation function with a temperature parameter is applied to the Generator’s last layer. In this sense, when sampling SMILES, the randomness of the probabilities associated with choosing the next token to integrate the molecule can be regulated by adjusting this temperature. Therefore, it is possible to control the model’s susceptibility to discover new actions.

Second, two agents having the same structure are integrated into the RL process, simultaneously. The only difference between them is the policy. In other words, we have two Generators with different internal parameters to predict the constituent tokens of the molecules alternately. The unbiased Generator in Figure 1A is the initially trained model to generate valid molecules and is focused on the exploration. The biased Generator in Figure 1B results from successive updates of the parameters and is more focused on the exploitation. The balance between using the two models is made through random numbers at each step and the establishment of thresholds. If the number generated is greater than the defined threshold, is used and vice-versa. There are three possible values for this threshold. The selected value depends on the most-recent reward evolution (increasing, decreasing or indefinite). Therefore, we can guarantee that the will be more likely to be selected if the reward increases. This strategy considers the goal of discovering promising molecules and the purpose of preserving the diversity of the compounds.

At last, we created an external memory that contains the last SMILES generated that is updated dynamically. Then, as new SMILES are generated, their diversity is calculated and compared with the molecules present in memory. If the average diversity is smaller than a given threshold, the reward is penalized. Thus, after performing this correction, the weights will be adjusted for the next step, and the Generator will be able to get out of possible relative minimums. In order to calculate the diversity, we computed the Tanimoto similarity (*T_s_*) using the RDKit tool. This similarity is computed by converting SMILES to ECFP3, which is a binary vector that is constructed so that the more similar two molecules are, the more elements are equal in the respective vectors. Therefore, the distance between two sets of molecules A and B, δ(A,B) is the average of the Tanimoto distance ($1-T_{s}$) of every pair of molecules from the sets *A* and *B* (Benhenda, 2017):

(6) $$\delta (A,B)=\frac{1}{\left|A|\cdot |B\right|}\sum_{a\in A}^{\left|A\right|}\sum_{b\in B}^{\left|B\right|}\left(1-\frac{\left|m_{a}|\cap |m_{b}\right|}{\left|m_{a}|\cup |m_{b}\right|}\right),$$

where $\left|m_{a}|\cap |m_{b}\right|$ indicate the coefficient between the number of matching bits and $\left|m_{a}|\cup |m_{b}\right|$ the total number of bits formed by the two compounds. Thus, *T_s_* between two molecules varies from 0 (less similar) to 1 (same molecule). The previously mentioned strategies aim to preserve the compounds’ diversity while optimizing their affinity for the target. The binding affinity is measured using the ${\text{pIC}}_{50}$, and the higher this value, the higher the potential of the molecule to be an antagonist inhibitor of the $A_{A2A}R$. Similarly as Liu *et al.* during the presentation of the results, a compound will be defined as *desirable* if the respective ${\text{pIC}}_{50}$ is greater than 6.5 Liu *et al.* (2019).

## 3 Datasets and results

### 3.1 Datasets

The Generator is trained with the SMILES compounds obtained from the Zinc database (Sterling and Irwin, 2015). The dataset included 499 915 SMILES with a logP ranging from -2 to 6, and, molecular weight between 200 and 600 g/mol. The affinity property Predictor is trained on a dataset of the ChEMBL (Mendez *et al.*, 2019), 4872 compounds, and their respective biological affinities for the $A_{A2A}R$ (ChEMBL identifier: CHEMBL251). Finally, BBB Predictor is implemented with a dataset composed of molecules collected from various works (Adenot and Lahana, 2004; Roy *et al.*, 2019; Wu et al., 2018; Zhao et al., 2007). The respective SMILES are canonicalized and formed a set of 4534 molecules.

### 3.2 Validity and analysis on QSAR models

The two Predictors integrated into the RL training process are conceptually different, and it is necessary to ensure the robustness of both to improve the reliability of subsequent results.

**Fig. 3.**
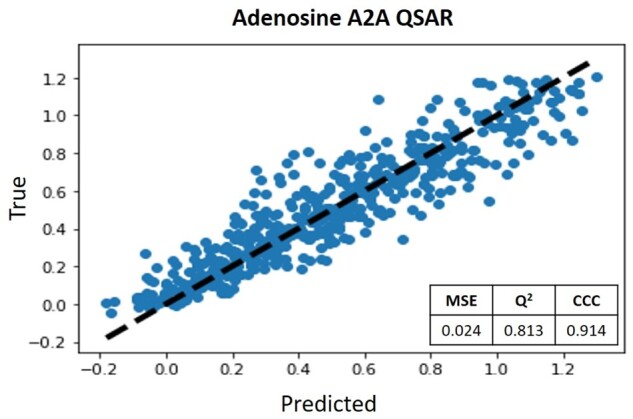
$A_{A2A}R$ QSAR scatter plot and evaluation metrics: MSE, *Q*^2^ and CCC

First, to estimate the compounds’ biological affinity for the $A_{A2A}R$, we implement a regression QSAR that maps SMILES strings to the desired property. Therefore, metrics such as Mean Squared Error (MSE), Coefficient of Determination (*Q*^2^) and Concordance Correlation Coefficient (CCC) were evaluated as they are applied in the literature (Gramatica and Sangion, 2016). Figure 3 summarizes the obtained results for the $A_{A2A}R$ Predictor. The *x*-axis shows the real biological activity values, and the *y*-axis shows the corresponding predictions of the proposed QSAR. The goal is to have the points as close as possible to the diagonal line. Therefore, both the evaluated metrics and the dispersion of the points close to the diagonal line confirm the model’s robustness for the entire range of biological affinity values. Thus, SMILES strings’ ability to be meaningful descriptors is proven when integrated with networks based on recurrent architectures.

Second, the permeability BBB Predictor goal is to predict whether compounds can cross the BBB or not. Therefore, the problem is a binary classification problem. However, as we apply oversampling techniques, it is necessary to evaluate the best molecular descriptor to calculate the distances between the compounds (a necessary step in oversampling) and the most suitable oversampling approach for this context. Table 1 depicts the results obtained in terms of Accuracy (ACC), Area Under Curve (AUC), Sensitivity (Sen), Specificity (Spe) and Mathews Correlation Coefficient (MCC).

**Table 1.** Comparison of different combinations of descriptor and oversampling technique for the implementation of the BBB QSAR.

Descriptor	Oversampling	Acc	AUC	Spe	Sen	MCC
SMILES	ADASYN	0.924	0.955	0.818	0.958	0.788
SMILES	SMOTE	0.913	0.928	0.737	0.952	0.722
ECFP	ADASYN	0.935	0.922	0.859	0.963	0.829
**ECFP**	**SMOTE**	**0.944**	**0.971**	**0.898**	**0.951**	**0.834**

The approach that yielded the best result is highlighted in bold.

The results on the Table 1 confirms that the molecules that can cross the BBB are more easily identifiable since sensitivity is higher than specificity in all experiments. This is an expected result as the initial dataset has far fewer examples of the BBB non-permeable molecules. Oversampling techniques aim to minimize this tendency through synthetic data generation but do not eliminate it entirely. This difficulty in identifying non-permeable molecules can be partially explained by the fact that the synthetically generated molecules were based on the initial non-permeable molecules. As such, they may not add information in quality but only in quantity. QSAR models with ECFP descriptors achieve better results than SMILES notation in terms of oversampling since they are binary vectors and, consequently, it may be easier to synthetically synthesize new molecules compared to SMILES notation. In other words, each token in SMILES is encoded by a number from 0 to 44. Therefore, the synthetic generation of new molecules of the underrepresented class is more complicated due to the higher sparsity of SMILES. Nonetheless, Table 1 indicates that ECFP and SMOTE is the best combination of the descriptor and oversampling technique, respectively, to implement the BBB Predictor.

### 3.3 Experimental analysis on the unbiased generator

The unbiased Generator is aimed to be as versatile as possible. In other words, although it does not have to optimize the molecules’ properties, it has to be evaluated in terms of validity rate, diversity and uniqueness. The analysis of these metrics was performed by generating 10 000 molecules, and the results are summarized in Table 2.

**Table 2.** Evaluation of Unbiased Generator

% desirable	% valid	Int. diversity	Ext. diversity	% Unique
86.3	93.1	0.91	0.91	99.9

The percentage of molecules that respect the RDKit parsing demonstrates that the Generator based on recurrent architectures and SMILES notation can learn the rules for constructing chemically valid molecules since roughly 93% are valid. Moreover, the remaining parameters indicate that the model can sample new molecules presenting diversity and novelty compared to the training dataset. These properties are fundamental for the success of the next step in which the fine-tune the biased Generator through RL.

### 3.4 Analysis on biased generator and multi-objective optimization reward

**Fig. 4.**
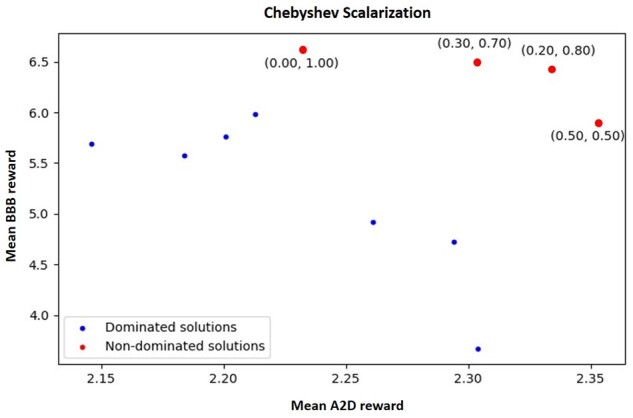
Dominated and non-dominated solutions obtained for linear weighted sum scalarization

**Fig. 5.**
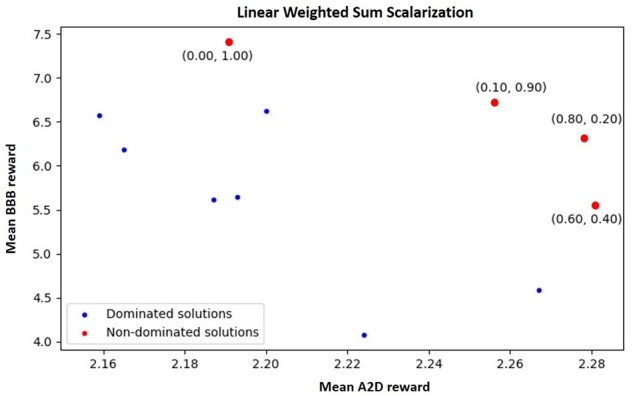
Dominated and non-dominated solutions obtained for Chebyshev scalarization

The parameters and hyper-parameters that optimize the performance of the RL and ensure the proper convergence of the loss function have been determined by grid-search. Thus, we trained the model for 400 episodes using Adam optimizer with a learning rate of 0.001. Gradients have been clipped to [-3,3] to avoid abrupt variations. The molecules had a maximum of 65 tokens, and each generated batch contained 10 molecules. For adjusting the exploration/exploitation dilemma, Softmax temperature set to 0.9, and the thresholds when the reward is increasing, decreasing or following an undefined trend are 0.01, 0.05 and 0.1, respectively. Finally, the conversion from the predicted *pIC*_50_ of the molecule to the assigned reward is performed using the following rule: $R_{t}$ = $\text{exp}(\frac{pIC_{50}}{4}-1)$. The integration of multi-objective optimization with RL aims to find molecular characteristics that allow the compounds to inhibit the $A_{A2A}R$ by maximizing the *pIC*_50_ value and cross the BBB. This combination is produced using two scalarization techniques that will transform the rewards of each property into a single numerical value. However, it is necessary to previously assign a weight for each objective in both techniques, between 0 and 1. The followed approach was to perform a uniform sampling of the weights between the two extremes with a step of 0.1. Figures 4 and 5 represent the solutions obtained for each weight assignment. Each solution is a point whose coordinates are the average rewards obtained in the last iterations of the RL process for each objective.

For both scalarization techniques, it is visible that the two objectives are competing. In other words, as we assign more importance (higher weight) to one of the objectives, the obtained reward at the end of the process is much more significant for the objective with greater weight compared to the other. Nonetheless, some unexpected results may be justified by the stochasticity of the model (it possesses some inherent randomness) and the influence of a third objective to be optimized that is not directly represented: the diversity of the compounds. Throughout the Generator’s fine-tuning process, situations may arise in which one of the objectives is optimized so strongly that the model enters a minimum of the loss function that only allows the generation of similar molecules. As previously mentioned, this repeatable generation will cause the reward to be penalized so that the model can get out of that minimum. However, this forced correction can also modify the expected result at the end of the optimization process (for one or both objectives), taking into account the earlier weight assignment.

The solutions highlighted in red are non-dominated policies, i.e. these solutions optimize both objectives better than the dominated solutions, represented in blue. Hence, it was impossible to find a single solution that is considered the best for the two objectives, at the same time. However, by comparing the possible pairs of non-dominated solutions for each scalarization technique, we can see that each solution has only one objective with a higher reward than the other. For such a reason, the solutions are incomparable, and it is necessary to carry out a more objective assessment to identify the solution that presents the most beneficial trade-off for this context.

In this sense, the most promising policies were compared through the characteristics of the respectively generated molecules. These characteristics include the Generator’s ability to skew the two desired properties while maintaining the validity, diversity and uniqueness in the generated compounds. Further, for the best configuration identified, we ran an additional experiment in which it was employed the same architecture but without the two-generator strategy to demonstrate its effectiveness. It is noticeable the decrease in the diversity and the uniqueness of the generated molecules. Table 3 summarizes the obtained results for two scalarization techniques: Chebyshev and Linear Weighted Sum (LWS).

**Table 3.** Comparison of the non-dominated solutions obtained for each scalarization technique.

Technique	Weight A2A	A2A biasing	BBB biasing	Valid (%)	Diversity	Unique (%)
Chebyshev	0	0.154	0.343	94.4	0.827	77.8
Chebyshev	0.2	0.503	0.239	83.2	0.769	77.9
Chebyshev	0.3	0.420	0.182	88.6	0.758	75.4
**Chebyshev**	**0.5**	**0.530**	**0.328**	**90.0**	**0.783**	**76.3**
Chebyshev*	**0.5**	**0.724**	**0.154**	**94.6**	**0.672**	**61.3**
LWS	0	0.199	0.225	85.6	0.795	84.1
LWS	0.1	0.277	0.352	76.6	0.819	77.1
LWS	0.6	0.313	0.201	87.8	0.807	85.3
LWS	0.8	0.280	0.121	79.4	0.801	83.9

*Note*: Chebyshev* relates to the experiment that was carried out without the use of the two-generator strategy.

The best scalarisation strategy is highlighted in bold.

**Fig. 6.**
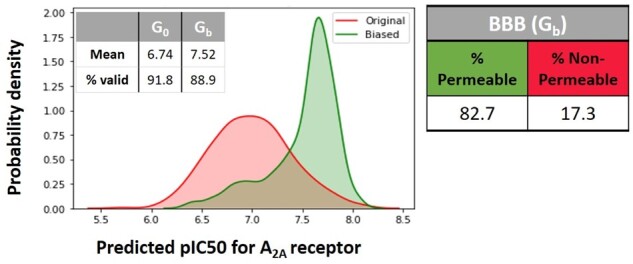
Comparison between unbiased and biased Generators regarding the *pIC*_50_ probability density for $A_{A2A}R$ and percentage of permeable molecules across BBB

Although the two objectives are conflicting, they are not antagonistic since it’s possible to identify some policies that ensure an appropriate trade-off between them. On that account, we can highlight the solution obtained through Chebyshev scalarization when we assigned 0.5 for the importance of both objectives. In this case, nearly 82.7% of the molecules have both properties skewed in the desired direction, and the percentage of syntactically valid molecules has remained at a reasonable level. These findings are comparable to the GCPN framework, in which the authors achieved on average 75% and 70% success in the molecules generated in the optimization of logP and MW, respectively. Figure 6 compares the outcomes of the unbiased and biased Generators according to the scalarization approach and weight assignment previously mentioned by generating 5000 compounds.

**Fig. 7.**
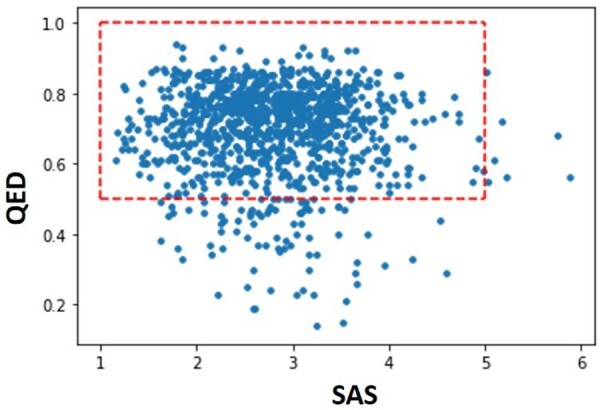
Evaluation of the QED and SAS score for the molecules obtained from the biased Generator. The desirable region for the properties is inside the red dashed lines

Hence, we can verify that the molecules generated by this policy are likely to simultaneously inhibit the $A_{A2A}R$ and penetrate across the BBB. However, in addition to optimizing competing properties as desired, we intend to demonstrate that this approach can generate synthesizable and drug-like compounds that are necessary conditions to consider the biased Generator’s outcome as promising lead molecules. In this sense, two important properties were evaluated: the Quantitative Estimate of Druglikness (QED) and Synthetic Accessibility Score (SAS). The first allows compressing the evaluation of diverse drug characteristics (such as octanol-water partition coefficient, number of hydrogen bond donors, number of hydrogen bond acceptors, molecular polar surface area, number of rotatable bonds, number of aromatic rings and number of structural alerts) in a single numeric value between 0 and 1 (Bickerton *et al.*, 2012). Maximizing this parameter guarantees drug-like molecules’ generation in terms of its absorption, distribution, metabolism, excretion and toxicity (ADME/tox). The second metric is a tool to evaluate the difficulty of synthesizing the molecules based on the knowledge extracted from known synthetic reactions and high molecular complexity penalization. This metric varies between 1 and 10, i.e. if a molecule has scored more than 6, it is considered challenging to synthesize (Ertl and Schuffenhauer, 2009). Figure 7 depicts the distribution of both properties for a set of 5000 molecules sampled from the biased Generator.

**Fig. 8.**
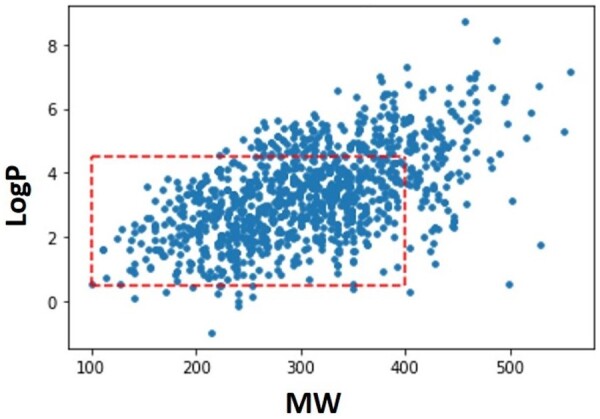
Evaluation of the logP and MW for the molecules obtained from the biased Generator. The desirable region for the properties is inside the red dashed lines

It’s noticeable for the two properties that the most considerable part of the molecules is located within the desired ranges. Therefore, it confirms their potential to be drug-like and synthesizable molecules. Regarding the BBB, we evaluate two properties that influence the molecules’ permeability: the MW and the logarithm of the partition coefficient between octanol and water (logP). Generally, CNS active molecules tend to have a lower MW. Namely, in order to cross the BBB by passive diffusion, the molecules must have an MW less than 500. Also, CNS active drugs must have appropriate lipophilicity to cross the hydrophobic phospholipid bilayer of cell membranes. This property can be estimated through the logP whose values should be range between 1 and 4. Theoretically, the higher the lipophilicity, the more likely the drug is to cross the non-polar phospholipid layer. However, excessively lipophilic compounds will have penalization in its solubility, increased metabolism and eventually negatively affect the compound’s BBB penetration (Kerns and Di, 2008). In other words, greater lipophilicity increases the non-specific binding in the brain and, as such, decreases the biological activity of the compound in the target of interest. Figure 8 shows the distribution of the molecules generated by the biased Generator for logP and MW.

**Fig. 9.**
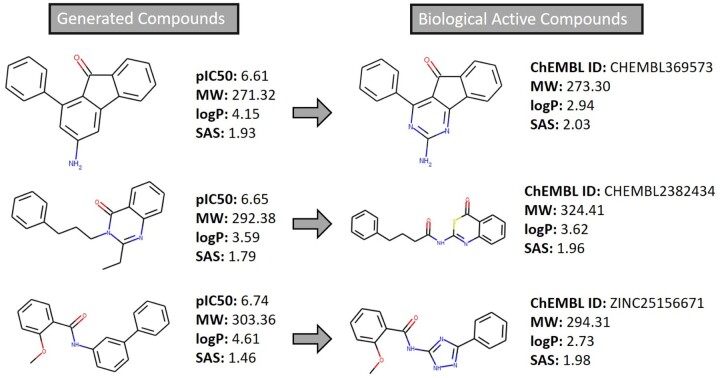
Comparison of generated molecules with similar biologically active compounds

In this case, the largest part of the molecules is also within the optimal range mentioned above for both properties. However, in the case of logP, we detect a high number of molecules with this parameter slightly above 4. This fact confirms that the biased Generator also identified that, to a certain extent, the increase in lipophilicity favours the permeability of the molecules. Figure 9 shows, on the left side, some compounds obtained from the biased Generator and their properties whose importance has already been discussed. On the right side, we represent molecules—with a high degree of similarity for each example of the biased Generator—that are known to interact with the $A_{A2A}R$. One of the possible weaknesses of this work is the possibility that Predictors are unable to indicate which actions are more favourable to create molecules with high biological affinity and BBB permeability due to having been trained with a restricted number of molecules. These similarities between molecules are an important validation of the framework as it demonstrates that it is possible to obtain novel compounds with a high degree of similarity with other molecules already identified as likely to bind to $A_{A2A}R$ and cross the BBB.

## 4 Conclusion

In summary, we have developed a *de novo* molecule generation framework to make the process of identifying lead molecules more effective. The novelty of our approach stems from its focus on creating molecules from the scratch that are likely to cross the BBB. Usually, computational generation methods consider the optimization of the drug–target affinity, disregarding the properties that influence its bioavailability. Hence, we were able to generate molecules with two essential optimized objectives: biological on-target affinity and permeability across BBB. As a result, it was computationally possible to get closer to the real complexity of this problem.

The implementation of this end-to-end framework involved the combination of several techniques such as DL, multi-objective optimization and RL. The backbone is the DL since the Generator and Predictors are based on artificial neural networks with several layers, which demonstrates the versatility and representativeness of this approach. Multi-objective optimization was essential to combine the two competing objectives accurately. Nevertheless, the cornerstone of this framework is the RL. Its application allowed us to update the properties of the sampled molecules as desired by maximizing a reward function.

The results demonstrate that in addition to having the two properties appropriately skewed, the molecules are synthesizable in the laboratory and present an interesting drug-likeness degree. These three factors are essential to consider the biased Generator a proper source of potential lead molecules. However, although they are encouraging, the results need definitive validation both in terms of affinity with the target and permeability at BBB. Future work will focus on the implementation of more robust predictor models to filter the newly generated compounds. At a later stage, we plan to explore 3D information of the compounds and the corresponding drug–target complexes to assess candidate drugs’ efficacy.

## Funding

This work was supported by the Portuguese Research Agency FCT, through D4 – Deep Drug Discovery and Deployment [CENTRO-01-0145-FEDER-029266].

*Conflict of Interest*: The authors declare that there is no conflict of interest.
